# Radial Artery Injury with Attempted Intravenous Catheterization on the Dorsal Hand: A Case Report

**DOI:** 10.7759/cureus.3033

**Published:** 2018-07-23

**Authors:** Yusuf Alimi, Joe Iwanaga, Rod J Oskouian, Marios Loukas, R. Shane Tubbs

**Affiliations:** 1 Anatomy, St. George's University School of Medicine, St. George's, GRD; 2 Medical Education and Simulation, Seattle Science Foundation, Seattle, USA; 3 Neurosurgery, Swedish Neuroscience Institute, Seattle, USA; 4 Anatomical Sciences, St. George's University, St. George, GRD; 5 Neurosurgery, Seattle Science Foundation, Seattle, USA

**Keywords:** radial artery, injury, intravenous, anatomy

## Abstract

Intravenous (IV) access on the dorsum of the hand is of high clinical significance. As it is possible for an arterial injury during IV access on the dorsum of the hand to occur, clinicians and healthcare providers should be cognizant of regional anatomy and anatomical variations during IV placement so as to prevent injuries to the radial artery. We report a case of an injury to the radial artery with an attempted hand IV placement in a cadaver and suggest possible ways to prevent this complication.

## Introduction

Venous access is of high clinical importance, as it allows for blood sampling as well as the administration of medications, fluids, nutrition, and chemotherapy [1]. The history of intravenous (IV) therapy can be traced back to the Middle Ages in the earliest attempts to transfuse blood [2]. It has since been a cornerstone of medical practice all over the globe, with almost every surgical patient requiring the placement of an IV catheter [3]. However, IV placement has not been without complications. The well-known complications include dysfunctional catheters and catheter-associated infections [4]. Injuries to peripheral nerves have been reported, along with thrombosis and phlebitis of the vessel involved [3-4]. Herein, we report a case of an injury to the radial artery, with attempted IV placement into the hand, found during cadaveric dissection.

## Case presentation

**Figure 1:**
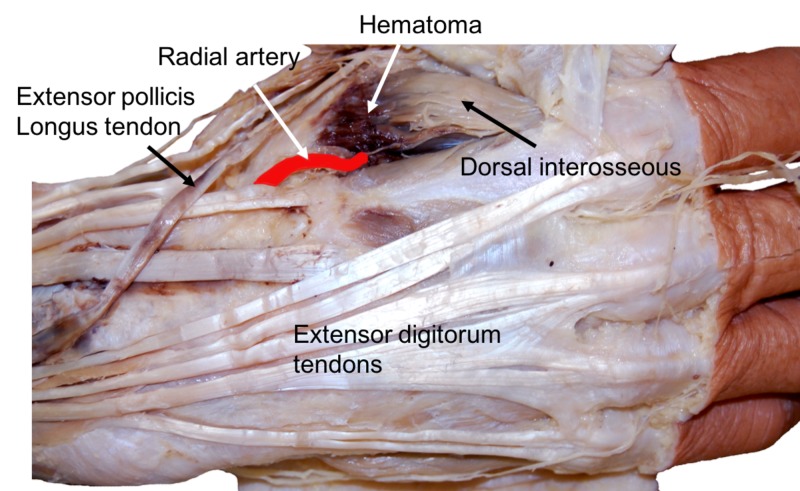
Right hand of the case presented herein Note the hematoma involving the first dorsal interosseous muscle and the relationship to the radial artery, which has been colored red for clarity.

During a routine dissection of the dorsal hand in a male cadaver, a large hematoma (Figure 1) was identified on the dorsal surface of the right hand. Both subcutaneous hematoma and a hematoma within the first dorsal interosseous muscle were found. The skin overlying this area was found to have tributaries of the cephalic vein within it. After the removal of the subcutaneous part of the hematoma, the radial artery, after traversing the anatomical snuffbox, was seen entering the two heads of the first dorsal interosseous muscle in the normal fashion. As the intramuscular part of the hematoma was adjacent to the radial artery, microdissection of this adjacent vessel was performed and a puncture site with thrombosis found in the adventitia of this vessel. Therefore, the diagnosis of radial artery puncture with resultant hematoma was made. No other disease or pathology of the hand was noted and no grossly visible anatomical variations were noted.

## Discussion

The radial artery originates from the brachial artery after the later bifurcates in the cubital fossa. This bifurcation is delineated by imaginary lines between the epicondyles superiorly, the medial border of the brachioradialis laterally, and the lateral border of the pronator teres muscle medially [5-6]. It then runs distally, taking a lateral path around the wrist and crossing the floor of the anatomical snuff box, which is typically bordered by the tendons of the extensor pollicis longus medially and the extensor pollicis brevis and abductor pollicis longus tendons laterally [5-6]. The cephalic vein runs on the dorsum of the hand, across the anatomical snuffbox, and is a common site of peripheral venous access and venipuncture [7-8]. Reports of atypical superficial branches of the radial artery in the hand, as well as its high origin from the brachial artery, are well documented [9-11] and since the dorsum of the hand is an area frequently used for IV access, a change in the normal course of the artery makes it susceptible to iatrogenic injury during these procedures [5,12].

One case report identified a patient with an atypical radial artery lodged superficially to the abductor and extensor pollicis longus tendons [12]. This superficial radial artery was mistaken for a vein due to its unusual anatomy. The patient underwent cannulation of the mistaken radial artery for post-operative IV administration [12]. Five days post cannulation, the patient presented with ecchymosis and darkening of the skin over the right thumb and index finger. It is well-established that an intra-arterial injection is a cause of pain, tissue necrosis, and ischemia at distal locations supplied by the vessel [13]. Physical examination findings indicated the loss of Doppler signals in the palm during ulnar artery occlusion (positive Allen’s test) and a purple appearance at the tip of the thumb, suggestive of a vascular injury to the radial artery [12]. An arteriogram showed the occlusion of the radial artery—secondary to thrombosis—and although thrombectomy and surgical repair of the radial artery were performed, the patient underwent amputation of the first two digits secondary to necrosis a month after the procedure [12]. Therefore, a good understanding of the anatomy and variants of the dorsal hand in regard to IV placement is essential for those placing such catheters [14-15].

## Conclusions

Although an understanding of the anatomy and variations of the radial artery are important when performing a venipuncture procedure on the dorsum of the hand, careful observation along with perioperative imaging studies (e.g., ultrasound) can help avoid complications. Injury to the radial artery can be minimized by palpating for a pulse before attempted catheterization and by not advancing the needle too deeply. Healthcare personnel should be aware of the possibility of injury to the radial artery and its surrounding neurovascular structures so as to prevent potentially serious and unwanted complications such as the large hematoma found in our case.
